# The correlation between food insecurity and infant mortality in North Carolina

**DOI:** 10.1017/S136898002200026X

**Published:** 2022-04

**Authors:** Lisa Cassidy-Vu, Victoria Way, John Spangler

**Affiliations:** Atrium Health Wake Forest Baptist Medical Center, Wake Forest School of Medicine, Department of Family and Community Medicine, Medical Center Blvd, Winston Salem, NC 27157, USA

**Keywords:** Nutrition, infant mortality, maternal health, social inequalities, health Inequalities

## Abstract

**Objective:**

Food insecurity (FI) affects approximately 11·1 % of US households and is related to worsened infant outcomes. Evidence in lower income countries links FI and infant mortality rates (IMR), but there are limited data in the USA. This study examines the relationship between FI and IMR in North Carolina (NC).

**Design:**

NC county-level health data were used from the 2019 Robert Woods Johnson Foundation County Health Rankings. The dependent variable was county-level IMR. Eighteen county-level independent variables were selected and a multivariable linear regression was performed. The independent variable, FI, was based on the United States Department of Agriculture’s Food Security Supplement to the Current Population Survey.

**Setting:**

NC counties.

**Participants:**

Residents of NC, county-level data.

**Results:**

The mean NC county-level IMR was 7·9 per 1000 live births compared with 5·8 nationally. The average percentage of county population reporting FI was 15·4 % in the state *v*. 11·8 % nationally. Three variables statistically significantly predicted county IMR: percent of county population reporting FI; county population and percent population with diabetes (*P* values, respectively, < 0·04; < 0·05; < 0·03). These variables explained 42·4 % of the variance of county-level IMR. With the largest standardised coefficient (0·247), FI was the strongest predictor of IMR.

**Conclusions:**

FI, low birth weight and diabetes are positively correlated with infant mortality. While correlation is not causation, addressing FI as part of multifaceted social determinants of health might improve county-level IMR in NC.

The US Department of Agriculture defines food insecurity (FI) as a condition of reduced quality and variety of food in the diet causing disrupted eating patterns and reduction in food intake^(1)^. Despite a gradual improvement in the percentage of Americans affected by FI over the last decade, in 2018, there was an estimated 11·1 % of American households reporting FI, which amounts to 14·3 million Americans lacking access to food^(2)^. The Covid-19 pandemic has highlighted the need to address FI. The crisis has exaggerated resource disparities and increased rates of FI, particularly in vulnerable populations already at risk for FI^(3–5)^.

Certain subgroups of Americans are more likely to be food insecure. FI is more prevalent in households below the 185 % federal poverty level (31·6 %); those headed by a single mother (31·6 %) or single father (21·7 %) *v*. those without children (8 %); and in black (22·5 %) and Hispanic households (18·5 %) *v*. white households (9·3 %)^(6)^. Rates of FI are also higher in families with adult smokers, those with disabled children or grandchildren, those with American Indians and those at risk of homelessness^(7–13)^. Residential segregation compounds these factors. One study found that there are 30 % fewer supermarkets in lowest income neighbourhoods^(14)^. In smaller stores within low-income neighbourhoods, food prices are higher with less selection and poorer quality of healthy foods^(14)^. A low-income, single mother may not have access to transportation to shop at a supermarket, or she may be limited by the time it takes to do so while working and caring for her children^(15)^.

FI has significant consequences for pregnant women and their unborn children. While definitions vary in the literature, FI is associated with greater weight gain and other prenatal complications, such as gestational diabetes mellitus^(16)^, and with poor intake of micro- and macronutrients^(17)^. During pregnancy, adequate intake of Fe and the fatty acid DHA improves fetal brain growth and neurodevelopmental outcomes^(18)^. Low folic acid stores are associated with neural tube defects, preterm delivery, intrauterine growth restriction and low birth weight (LBW)^(19,20)^. In a recent study of ninety-five lower income countries, decreased access to food was related to an increase in undernourishment and overall increase in infant mortality (IM)^(21)^. In rural Gambia, increasing the intake of protein, Ca and Fe led to an increase in birth weight and decreased perinatal mortality^(22)^. Apart from LBW and IM, birth defects from FI include tetralogy of Fallot, transposition of the great arteries, cleft palate and even anencephaly^(23)^.

Outside of the perinatal period, FI affects matriarchal heads of households. The mother may self-reduce her food intake to prevent hunger in children residing in the home^(24)^. FI negatively influences initiation and duration of breastfeeding^(25)^. FI is associated with decreased well-being of entire households; including higher rates of maternal depression and higher rates of family conflict^(26)^. Maternal depression from FI can lead to neglect of her children by reducing her ability to shop for food and care for herself and her children^(27)^. This can exacerbate the lack of food available to her children.

Although it is well known that FI in lower income countries is a significant factor in IM^(28–32)^, there are less data in the USA since the development of the Women, Children and Infant nutritional program. When instituted in the 1970’s, Women, Children and Infant showed a decrease in IM^(33)^. Research shows that the current leading causes of IM in the USA are due to congenital malformations, preterm delivery and LBW^(34)^. Since FI is linked to PTD and LBW in lower income countries^(35–37)^, it might be argued that FI has the same influence in the USA. However, since the initiation of Women, Children and Infant, FI in the USA and the impact on IM has not been evaluated in a meaningful way in a state such as North Carolina (NC) that did not expand Medicaid under the Affordable Care Act. This study will aim to examine the relationship between FI and IM in NC specifically.

## Methods

We used NC county-level health data from the 2019 County Health Rankings^(38)^, a database of the nation’s 3000 plus counties assembled and annually updated by the University of Wisconsin Population Health Institute and the Robert Wood Johnson Foundation. The County Health Rankings include both health outcomes such as morbidity and mortality rates; and health factors such as health behaviours, clinical care and socio-economic factors. Data are derived from publically available datasets including the National Center for Health Statistics Mortality and Natality files, the Behavioral Risk Factor Surveillance System, Map the Meal Gap from Feeding America, the American Community Survey, the Current Population Survey, the Census Bureau and others. A full list of 2019 County Health Ranking measures and sources is available at: https://www.countyhealthrankings.org/explore-health-rankings/measures-data-sources/2019-measures ^(39)^. All data exists in the public domain and therefore the study is exempt from institutional review board review.

We were particularly interested in the relationship between infant mortality rates (IMR) and FI. We used IMR from the North Carolina State Center for Health Statistics, defined as the number of deaths of infants under 12 months of age per 1000 live births for the combined years 2011–2017, a 6-year range because infant death is a rare event for many counties^(40)^. FI was defined as the percentage of the population who lack adequate access to food. The Economic Research Service of the United States Department of Agriculture derives this composite measure. It is based on answering affirmatively to three out of eighteen questions on Food Security Supplement to the Current Population Survey in 2018^(1)^. This survey includes such household questions as ‘We worried whether our food would run out before we got money to buy more’; ‘In the last 12 months, were you ever hungry, but didn’t eat, because there wasn’t enough money for food?’; and ‘. In the last 12 months, were the children ever hungry but you just couldn’t afford more food?’ (see online supplementary material, Supplemental material 1).

Multiple regression analysis was employed to predict 2011–2017 county-level IMR (the dependent variable) using eighteen county characteristics as independent variables from the 100 NC counties (Table 1). These variables were selected because of their importance to IMR as mentioned in the literature. The county-level independent variables comprised socio-demographics (population size, race/ethnicity, uninsured, college education, household income), health status (diabetes and obesity prevalence), health behaviours (smoking, excessive drinking, physical inactivity) and health outcomes (teenage pregnancy rates, LBW). We selected dates of independent variables to be as close to 2017 as possible so that any policy implications of the analysis would be more current. To normalise distributions, percent population by race/ethnicity and population per square mile were transformed to the natural log for analysis. Four counties (Davie, Greene, Hertford and Swain) were excluded from the data because their residuals were ≥ 3 standard deviations from the mean which made the distribution of the residuals non-normal. Running the analysis with and without these counties did not materially change the regression coefficients. In multiple regression, we first carried out bivariate analysis for all independent variables and IMR. Then variables were excluded from or included into the model stepwise based on the probability of F > 0·20 and < 0·10. Visual inspection showed homoscedasticity based on the dependent variable’s P–P plot of expected-to-observed cumulative probabilities of IMR; and the scatter plot of regression standardised value of IMR to regression standardised residual value. The data were not multicollinear (tolerance > 0·1 and Variable Inflation Factor < 10). Statistical significance was set at *P* < 0·05. All analyses were carried out with IBM SPSS®, version 26.

**Table 1:** Averaged characteristics of NC’s 100 counties (se) along with corresponding US national rates

County characteristic	Averaged rates of NC counties (*n* 100)	se	US rates
IMR, 2011–2017*	7·9	0·21	5·8
Population of county 2017	105 996	17 083	NA
Percent population African American 2017**	20·2	1·6	12·3
Percent population American Indian 2017**	1·7	0·4	0·7
Percent population Hispanic 2017**	7·4	0·4	18·1
Percent population white non-Hispanic 2017**	68·0	1·8	60·6
Population per square mile, 2016**	195	27·3	75·2
Median household income in dollars, 2017	47 333	913	61 372
Percent uninsured adults, 2017	16·7	0·3	7·9
Primary care physicians per 100 000 population, 2017	53·4	3·2	
Percent adult population 25 years or older with some college, 2013–2017	58·3	0·9	60·8
Percent adult population reporting FI, 2017***	15·4	0·4	11·8
Percent population with diabetes, 2016	13·2	0·4	8·5
Number of births per 1000 female population ages 15–19, 2011–2017	31·6	1·0	18·8
Percent births < 2500 g, 2011–2017	9·3	0·2	8·3
Percent adult population with obesity, 2016	33·7	0·6	31·6
Percent adult population who smoke, 2017	17·4	0·2	17·4
Percent adult population with physical inactivity, 2016	27·7	0·5	23·1
Percent adult population who drink excessively, 2017	15·8	0·2	18·0

NC, North Carolina; IMR, infant mortality rate; FI, food insecurity.

* Per 1000 live births.

** To normalise distributions, the natural log of these variables were used in the regression equation.

*** FI defined as household-level economic and social conditions of limited or uncertain access to adequate food.

## Results

Averaged characteristics of NC’s 100 counties are listed in Table 1 along with corresponding US national rates. Compared with the national 2017 demographic composition, the state’s counties have a higher percent population of African American (20·2 % *v*. 12·3 %), American Indian (1·7 % *v*. 0·7 %) and non-Hispanic white (68 % *v*. 60·6 %). Conversely, there is a lower average percent of Hispanics in NC counties (7·4 % *v*. 18·1 %). The county-level median household income ($47 333) is lower than American households ($61 372) as is the percentage of adults 25 years or older who reported some college (2013–2017 rates: 53·4 % in NC *v*. 60·8 % in the USA). Mean county population per square mile is 195, but ranged greatly from 4 (Hyde County) to 1932 (Mecklenburg County). Granville County holds the state’s median population residents per square mile (109).

All NC county health-related variables in Table 1 except excessive drinking are less favourable (‘less healthy’) compared with the US population (NB: these are for illustrative purposes only since cannot derive inferential statistics without full US datasets). Contrasting with national averages, the average percent of county population reporting FI in 2017 is 15·4 % in the state *v*. 11·8 % in the American population. In 2017, NC counties have a higher percent of uninsured adult residents (16·7 % in NC *v*. 7·9 % in the USA) and diabetes (13·2 % in NC *v*. 8·5 % in the USA). The 2011–2017 mean county-level IMR is 7·9 deaths per 1000 live births in NC compared with 5·8 nationally. Two adverse gestational characteristics—teenage pregnancy rate and LBW—are higher in the state’s counties: Average county and national teenage birth rate 31·6 *v*. 18·8 births per 1000 teenage women, respectively; and average percent of LBW (< 2500 g) 9·3 % *v*. 8·3 %, respectively. Compared with other Americans, more NC adults smoke (17·4 % *v*. 13·7 % in 2017); are obese (33·7 % *v*. 31·6 %, 2016) and are physically inactive (27·7 % *v*. 23·1 %, 2016). Fewer NC adults by county drink excessively in 2017 compared with US rates (15·8 % *v*. 18·0 %).

Table 2 shows the multivariable regression analysis. Four variables remain in the equation, three of which are statistically significant: average percent of county population reporting FI in 2017; average county population in 2017 transformed to the natural log and percent population with diabetes in 2016 (p values, respectively, < 0·04; < 0·05; < 0·03). Percent LBW (< 2500 g) between 2011 and 2017 approaches statistical significance (*P* = 0·086). County level FI, diabetes and LBW are positively associated with IM. Each regression coefficient indicates how much the predicted value of the dependent variable (IMR) changes (increases or decreases) each time the dependent variable (FI, diabetes or LBW) changes (increases or decreases) by one unit^(41)^. Theoretically, for each 1 % increase (or decrease) in county-level FI, there will be an increase (or decrease) of 0·12 infant deaths per 1000 live births between 2011 and 2017; 10 % increase (or decrease) in county FI will add (or subtract) 1·2 additional infant deaths per 1000 live births for the time period. Population, log transformed, is negatively associated with IM. A 1 % increase in population yields approximately a 0·003 decrease in IM; and a 10 % increase in population yields 0·03 decrease in IM. This four variable model explains 42·4 % of the variance in IMR from 2011 to 2017 (adjusted R^2^ = 0·424). FI is a robust predictor, remaining in models even when limiting data from County Health Ranking years to 2013–2017. The standardised coefficient of FI (Beta = 0·247) has the largest absolute value indicating its importance compared to the three other variables. This value implies that for each one sd increase (or decrease) in FI by county, there will be a corresponding increase (or decrease) in county-level IM of 0·247 sd of deaths per 1000 live births.

**Table 2:** Regression analysis of county-level predictors of county-level IMR in NC counties (*n* 100)

Model	B	se	Adjusted Beta	*P* value
Percent population reporting FI, 2017*	0·118	0·056	0·247	< 0·04
Ln (population 2017)**	−0·290	0·144	−0·179	< 0·05
Percent population with diabetes, 2016	0·209	0·092	0·241	< 0·03
Percent births < 2500 g, 2011–2017	0·245	0·141	0·208	0·086

IMR, infant mortality rate; NC, North Carolina; FI, food insecurity.

* FI defined as household-level economic and social conditions of limited or uncertain access to adequate food.

** To normalise distribution, the natural log of this variable was used in the regression equation.

As mentioned, multivariate analysis confirmed that FI (Table 2) was the strongest correlate of IMR. The initial and final models displaying the unstandardised coefficients and their se are shown in Supplemental Table 2. The absolute value of FI unstandardised coefficient decreased when the final model controlled for all variables (0·265 ± 0·169 to 0·118 ± 0·056), as did the absolute values of the unstandardised coefficients for percent population with diabetes and percent LBW births (0·327 ± 0·183 to 0·209 ± 0·092; and 0·325 ± 0·221 to 0·245 ± 0·141, respectively). By contrast, the absolute value of the unstandardised coefficient for county population increased (–0·040 ± 0·53 to –0·290 ± 0·144).

## Discussion

Four county-level variables in NC explain a substantial amount of the variance (42·4 %) in the 2011–2017 IMR—percent of population experiencing FI, LBW, rate of diabetes, and low total population of each county. Apart from population, which has a negative relationship with IM, the other variables positively correlate with IM such that increases or decreases in these variables also imply increases or decreases in IMR.

Moreover, of the four variables remaining in the model in our study, FI has the strongest standardised coefficient (Beta = 0·247). Even though county-level data cannot be extrapolated down to specific infants or pregnant women, the data can be applied to county policy for each of the counties. Indeed of the four variables, FI is theoretically the most modifiable. Food assistance can be given directly to populations suffering from FI. It is known that FI is inversely related to food quality and food assistance programs such as community-based meal delivery services, SNAP and Women, Children and Infant decrease FI^(42–46)^. Could ensuring adequate food supply in NC’s counties lower county-level IMR?

These results are largely consistent with the current literature on IMR. While our study demonstrates that counties with a higher rate of diabetes in the population in general also have a higher rate of IM, the current data on this subject support the link specifically between maternal diabetes and IMR. For instance, a study done in Indonesia showed that diabetes during pregnancy may lead to an increased risk of IM^(47)^, though it is worth noting that with adequate control and prenatal care, the outcomes for premature/LBW infants born to mothers with diabetes are not significantly different than those with non-diabetic mothers^(48,49)^.

The relationship between IM and LBW is well studied, and our results are overall consistent. When evaluating IMR in Delaware (a state historically with a higher IMR than the national average), IM is linked to very LBW^(50)^. In the USA as a whole from 1983 to 2005, there is a very strong correlation between very LBW and IMR^(51)^. While the rates of IM overall decreased during that period, it is speculated that the number of LBW infants born prevents the rate of decline from being more drastic^(52)^. Although our data do not show a significant difference between races, there are data to suggest that the African American community is more likely to experience LBW and thus higher IM compared with the white community^(52)^.

As in our study, IMR are much higher in areas considered non-metropolitan compared with metropolitan areas^(52)^. Risk factors (maternal obesity, maternal smoking) for higher IMR appear to be more commonly found in non-metropolitan, or rural, areas. Rates are also higher in areas with decreased access to healthcare resources^(53)^. These factors may be confounding.

Our finding that FI positively correlates with increased IM in NC supports the prior findings of multiple studies largely performed in lower income countries. In nations ranging from Niger to Nepal^(28,54,55)^, FI is related to both IMR and mortality for children under 5 years^(30,31,56)^. Moreover, supplemental feeding and micronutrient provisions decrease IMR^(54)^. For example, in Nepal in 2003, it was found that Fe and folic acid deficiencies are thought to play a significant role in IM^(54)^, as supplementing mothers with these vitamins decreases IMR when compared with the control group of vitamin A supplementation alone^(56)^. Among pregnant women in poor communities in Bangladesh, treatment with multiple micronutrients and early food supplementation results in decreased childhood mortality^(21)^.

While not widely studied in the USA, our data reflect global trends. More than one-third of child deaths are due to maternal and/or child undernutrition^(57)^. It is estimated that effects of FI such as stunting, severe wasting and intrauterine growth restriction are jointly the cause of 2·2 million deaths and 21 % of disability-adjusted years of life for children younger than 5 years globally in the year 2005 alone. Vitamin A and Zn deficiencies are estimated to be responsible for 0·6 million and 0·4 million deaths, respectively. While Fe deficiencies in infants do not cause many deaths, lack of sufficient maternal Fe added 115 000 deaths in a single year^(57)^. It is not an unreasonable deduction to draw a link between the vitamin deficiencies listed above and food insecure status.

There is a scarcity of studies in the USA on FI and its effects on IM. As mentioned, there are several studies focussed on lower income countries, and our findings are congruent with these. We think it important to initiate a body of literature for FI in the USA and its relationship to IM, which is the impetus for this study.

As health professionals, we have to be more cognizant of the health determinants that affect our communities. On a community level, there are many possible solutions to the FI crisis. Residential segregation and zoning should be examined. Supermarkets with better accessibility to families can reduce LBW^(58)^. Supporting community gardens and local farmers markets can help with accessibility and affordability of fresh fruits and vegetables. Encouraging policies that mandate higher minimum wages can help impoverished families with transportation and location. Nationwide, we need a system in place that prompts us to ask our patients and clients about FI with readily available resources and solutions.

Our study is not without limitations. First, this information in this study is unique to NC and may not be generalisable outside the state. NC does not represent the entire country. We only assessed the relationship of IM to eighteen variables that are hypothesised to have clinical significance, though there are many other variables that are not addressed that may have shown a significant difference. These include but are not limited to illicit drug use, maternal comorbid medical conditions apart from diabetes and infant medical conditions/congenital malformations. There are several variables assessed that surprisingly do not show a clinically significant relationship with IMR. Specifically, smoking and race, which is somewhat conflicting with the current literature. Because of the type of data used (population level), there is potential for overshadowing and for confounding factors to limit the strength of the results.

## Conclusion

This study shows that FI is a potential predictor of IM. FI is also a serious public health issue and needs to be addressed as such. Our paper provides justification to study FI at the national level not only to see if our NC findings are validated, but also to determine if such findings can be translated by societal interventions that actually reduce IMR. Therefore, more research still needs to be done to look not only at FI and at its relationship to IMR, but also its relationship to other social determinants of health.

## Supplementary material

For supplementary material accompanying this paper visit https://doi.org/10.1017/S136898002200026X. click here to view supplementary material
