# Supplementary material for: The correlation between food insecurity and infant mortality in North Carolina
Source: Public Health Nutr. 2022 Jan 31;25(4):1038–44. doi: 10.1017/S136898002200026X (PMC9991794; doi:10.1017/S136898002200026X)
Supplement: Supplementary file 1 [file S136898002200026Xsup.zip › S136898002200026Xsup001.docx]

Supplemental Material 2: Unadjusted coefficients in initial and final models.

| **County Characteristic** | **Initial Model** | | **Final Model** | |
| --- | --- | --- | --- | --- |
|  | **B** | **SE** | **B** | **SE** |
| Percent Adult Population Reporting FI, 2017*** | 0.265 | 0.169 | 0.118 | 0.056 |
| Population of County 2017 | -0.040 | 0.553 | -0.290 | 0.144 |
| Percent Population with Diabetes, 2016 | 0.327 | 0.183 | 0.209 | 0.092 |
| Percent Births < 2500 Grams, 2011-2017 | 0.325 | 0.221 | 0.245 | 0.141 |
| Percent Population African American 2017** | 0.500 | 0.371 |  |  |
| Percent Population American Indian 2017** | 0.645 | 0.364 |  |  |
| Percent Population Hispanic 2017** | 0.773 | 0.662 |  |  |
| Percent Population White Non-Hispanic 2017** | 0.928 | 1.932 |  |  |
| Population Per Square Mile, 2016** | 0.646 | 0.497 |  |  |
| Median Household Income in Dollars, 2017 | 0.000 | 0.000 |  |  |
| Percent Uninsured Adults, 2017 | -0.125 | 0.174 |  |  |
| Primary Care Physicians per 100,000 Population, 2017 | -0.001 | 0.009 |  |  |
| Percent Adult Population 25 Years or Older with Some College, 2013-2017 | -0.071 | 0.044 |  |  |
| Number of births per 1,000 Female Population Ages 15-19, 2011-2017 | -0.073 | 0.031 |  |  |
| Percent Adult Population with Obesity, 2016 | -0.302 | 0.098 |  |  |
| Percent Adult Population who Smoke, 2017 | -0.348 | 0.185 |  |  |
| Percent Adult Population with Physical Inactivity, 2016 | 0.235 | 0.092 |  |  |
| Percent Adult Population who Drink Excessively, 2017 | 0.306 | 0.182 |  |  |

*Per 1000 live births

**To normalize distributions, the natural log of these variables were used in the regression equation.

***FI defined as household-level economic and social conditions of limited or uncertain access to adequate food.
