# Supplementary material for: The correlation between food insecurity and infant mortality in North Carolina
Source: Public Health Nutr. 2022 Jan 31;25(4):1038–44. doi: 10.1017/S136898002200026X (PMC9991794; doi:10.1017/S136898002200026X)

**The Correlation between Food Insecurity and Infant Mortality in North Carolina**

Supplemental Material 1: Food Security Questionnaire


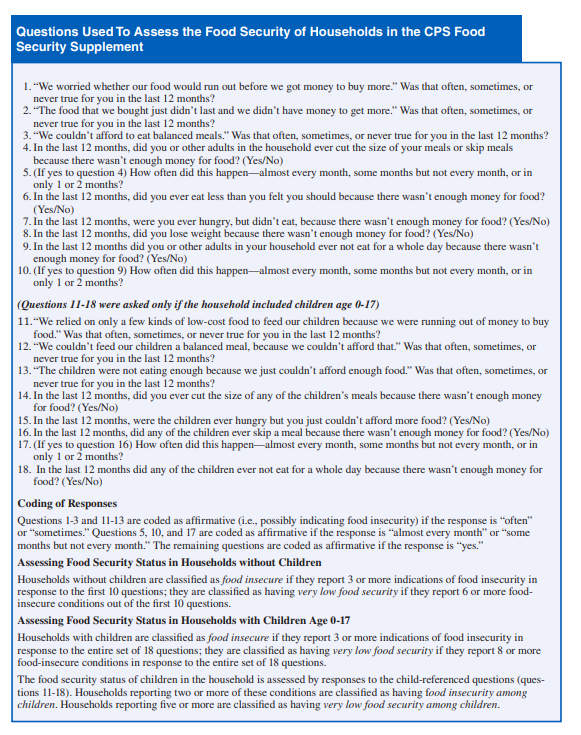

Supplement: Supplementary file 1 [file S136898002200026Xsup.zip › S136898002200026Xsup002.docx]
